# Empowering Minds: A Comprehensive Study of ECT Treatment in a Reference Mental Health Center in Portugal

**DOI:** 10.1192/j.eurpsy.2024.511

**Published:** 2024-08-27

**Authors:** M. Barbosa Pinto, M. T.D. Viseu, P. Frias Gonçalves, E. Gomes Pereira

**Affiliations:** ^1^CHUA, Faro; ^2^HML-CHUSA , Porto, Portugal

## Abstract

**Introduction:**

Electroconvulsive Therapy (ECT) is one of safest and most effective treatments for severe mental illnesses. The ECT Unit of Centro Hospitalar Universitário de Santo António – Magalhães Lemos Hospital (CHUSA-HML) is a reference center for this treatment modality, providing support to the northern region of Portugal.

**Objectives:**

This study aims to characterize patients undergoing ECT treatment from April to June 2023, at the ECT Unit of CHUSA-HML.

**Methods:**

Retrospective study from April to June/2023. Social, demographic, epidemiological and clinical data were evaluated.

**Results:**

Among the 55 patients who were treated there was a predominance in male sex (56%), the average age was 53 years old and only 9 completed higher education. Half of them were in a long-term relationship. Around 67% of patients are retired, predominantly (62%) due to psychiatric disability.

Most patients (78%) were referred through psychiatric consultation and the remainder came from psychiatric hospitalization (only 3 were never hospitalized). 41 patients were under maintenance treatment and 14 under acute treatment. Concerning the type of treatment 30 were submitted to bilateral ECT. For 33% it wasn’t the first ECT treatment. Almost all patients improved their symptoms, only one patient had complications related to the procedure (tooth loss).

According to the international classification of disease (ICD11) the most frequent primary diagnosis was Schizophrenia or Other Primary Psychotic Disorders (58%). Neurodevelopmental disorders and substance use disorders were the most frequently comorbid diagnoses.

The results presented are preliminary, and other data that may be relevant are being collected and processed.

**Conclusions:**

Severe mental illnesses profoundly impact patients, often imposing substantial limitations and suffering. These findings support the safety and effectiveness of ECT as treatment for severe mental disorders. Founding more specialized centers represents an important step toward enhancing mental health treatments. Access to controlled studies is crucial, fostering a deeper understanding of the ECT technique and long-term benefits.

**Disclosure of Interest:**

None Declared

